# P-2243. Multiplex PCR Pneumonia Panel in Critically Ill Patients Did Not Modify Mortality. A Cohort Study

**DOI:** 10.1093/ofid/ofae631.2396

**Published:** 2025-01-29

**Authors:** Luisa Fernanda Riaño-Sanchez, Jorge Cortes, Carlos Álvarez-Moreno

**Affiliations:** Universidad Nacional de Colombia, Bogota, Colombia, Distrito Capital de Bogota, Colombia; Universidad Nacional de Colombia, Bogota, Colombia, Distrito Capital de Bogota, Colombia; Universidad Nacional de Colombia, Clínica Universitaria Colombia, Bogota, Distrito Capital de Bogota, Colombia

## Abstract

**Background:**

Severe pneumonia is a common cause of Intensive Care Unit (ICU) admissions. In critically ill patients, identification of the pathogen may allow timely adjustment of antibiotics and, therefore improve outcomes. The aim of this study was to assess whether performing a multiplex polymerase chain reaction (PCR) pneumonia panel in patients with pneumonia in ICU, has any effect on mortality, overall stay, ICU stay and duration of antimicrobial therapy.

**Figure:**
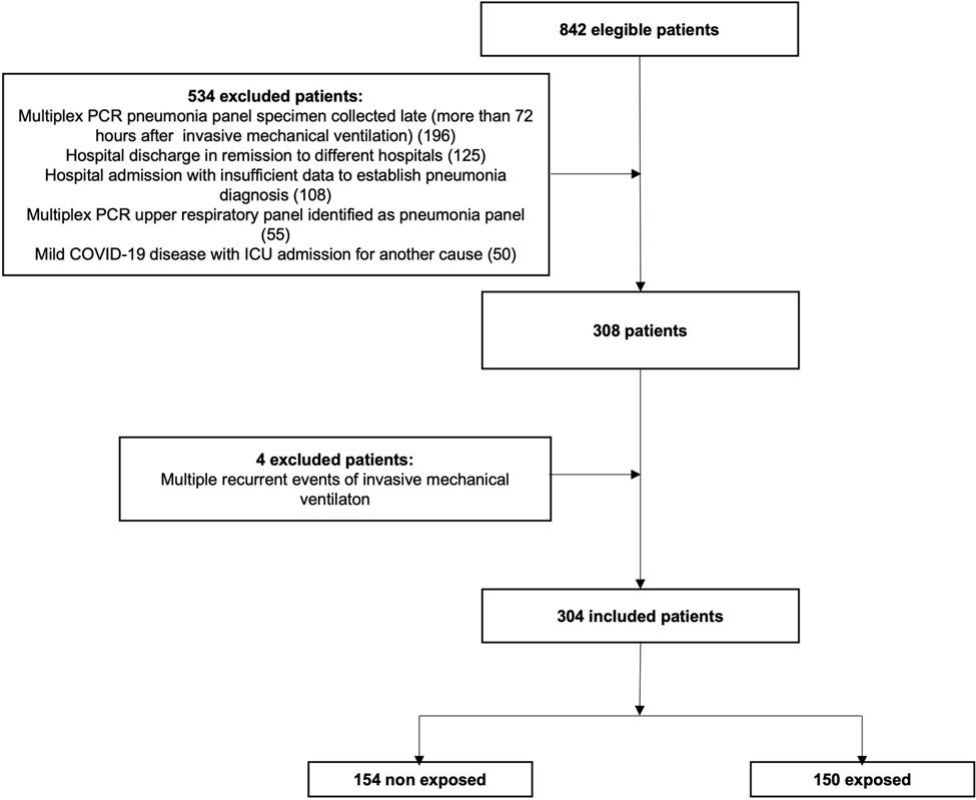

**Methods:**

A retrospective cohort study was conducted on adult patients with pneumonia who required ICU admission at 4 hospitals in Bogota Colombia, between November 2019 and June 2023. Patients who underwent multiplex PCR pneumonia panel testing were defined as exposed. Mortality at 30 days, length of hospital and ICU stay, duration of antibiotics and, their association with multiplex PCR pneumonia panel testing were evaluated using and Inverse Probability of Treatment Weighting (IPTW) to adjust for covariates and potential confounders. The infectious diseases specialist’s assessment and its effect on antibiotics adjustment was also determined.

Mortality analysis

**Figure:**
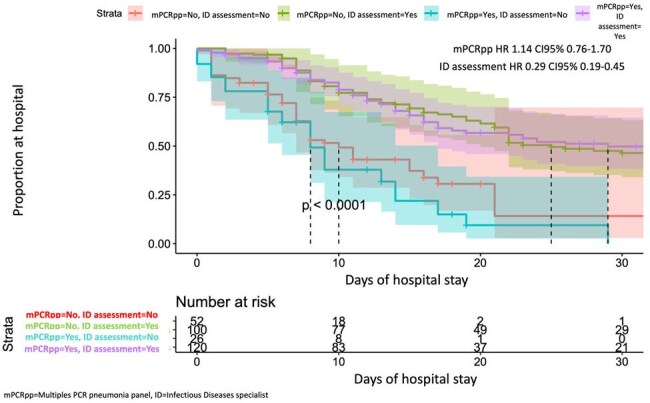

**Results:**

304 patients were included, 150 exposed, 154 non-exposed. Mean age 65.0 years (SD 14.6) and mean Charlson index 4.5 (SD 2.8). 186 (61.2%) patients had COVID-19 and 256 (84.2%) community acquired pneumonia. Length of hospital stay was 20.8 days (SD 17.0), ICU stay was 12.2 days (SD 10.9), total number of antibiotics administered was 2.7 (SD 1.7). No association was found between 30 days mortality and exposure HR 1.14 (CI95% 0.76-1.70), although infectious diseases specialist’s assessment is associated with a lower mortality HR 0.29 (CI95% 0.19-0.45). There was no association between exposure and antimicrobial therapy duration IRRa 1.17 (CI95% 0.90-1.54), hospital stay IRRa 1.02 (CI95% 0.75-1.38) or ICU stay IRRa 1.24 (CI95% 0.8-1.9).

Adjusted outcomes

**Figure:**
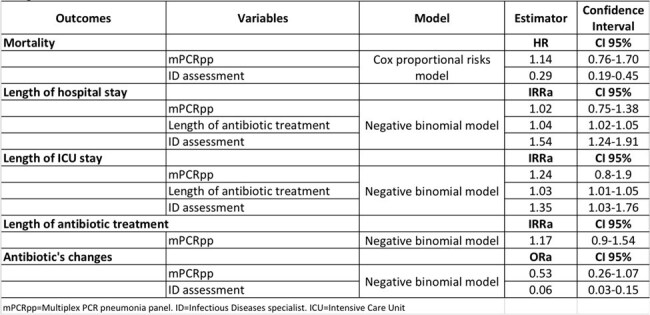

**Conclusion:**

The use of multiplex PCR pneumonia panel was not associated with changes in mortality, antibiotics duration, hospital or ICU stay. Integrating this type of testing into antimicrobial stewardship programs is necessary to contribute to more rational decision-making.

**Disclosures:**

Jorge Cortes, MD, Pfizer: Grant/Research Support

Carlos Álvarez-Moreno, PhD, Pfizer: Grant/Research Support

